# Supplementary material for: Efficacy of Zinc Fortified and Fermented Wheat Flour (EZAFFAW): A randomized controlled trial protocol
Source: PLoS One. 2024 Jun 20;19(6):e0304462. doi: 10.1371/journal.pone.0304462 (PMC11189184; doi:10.1371/journal.pone.0304462)
Supplement: S1 Checklist — (DOCX) [file pone.0304462.s001.docx]

SPIRIT 2013 Checklist: Recommended items to address in a clinical trial protocol and related documents*

| Section/item | ItemNo | Description | Page no |  |  |
| --- | --- | --- | --- | --- | --- |
| **Administrative information** | | |  |  |  |
| Title | 1 | Descriptive title identifying the study design, population, interventions, and, if applicable, trial acronym | 1 |  |  |
| Trial registration | 2a | Trial identifier and registry name. If not yet registered, name of intended registry | 5 |  |  |
|  | 2b | All items from the World Health Organization Trial Registration Data Set | Yes |  |  |
| Protocol version | 3 | Date and version identifier | Yes – in the protocol |  |  |
| Funding | 4 | Sources and types of financial, material, and other support | 1 |  |  |
| Roles and responsibilities | 5a | Names, affiliations, and roles of protocol contributors | 1 |  |  |
|  | 5b | Name and contact information for the trial sponsor | 1 |  |  |
|  | 5c | Role of study sponsor and funders, if any, in study design; collection, management, analysis, and interpretation of data; writing of the report; and the decision to submit the report for publication, including whether they will have ultimate authority over any of these activities | 1 |  |  |
|  | 5d | Composition, roles, and responsibilities of the coordinating centre, steering committee, endpoint adjudication committee, data management team, and other individuals or groups overseeing the trial, if applicable (see Item 21a for data monitoring committee) | yes |  |  |
| Introduction |  |  |  |  |  |
| Background and rationale | 6a | Description of research question and justification for undertaking the trial, including summary of relevant studies (published and unpublished) examining benefits and harms for each intervention | 3-4 |  |  |
|  | 6b | Explanation for choice of comparators | 3-4 |  |  |
| Objectives | 7 | Specific objectives or hypotheses | 4-5 |  |  |
| Trial design | 8 | Description of trial design including type of trial (eg, parallel group, crossover, factorial, single group), allocation ratio, and framework (eg, superiority, equivalence, noninferiority, exploratory) | 5 |  |  |
| Methods: Participants, interventions, and outcomes | | |  |  |  |
| Study setting | 9 | Description of study settings (eg, community clinic, academic hospital) and list of countries where data will be collected. Reference to where list of study sites can be obtained | 8-9 |  |  |
| Eligibility criteria | 10 | Inclusion and exclusion criteria for participants. If applicable, eligibility criteria for study centres and individuals who will perform the interventions (eg, surgeons, psychotherapists) | 7-8 |  |  |
| Interventions | 11a | Interventions for each group with sufficient detail to allow replication, including how and when they will be administered | 5-6, 11-13 |  |  |
|  | 11b | Criteria for discontinuing or modifying allocated interventions for a given trial participant (eg, drug dose change in response to harms, participant request, or improving/worsening disease) | y |  |  |
|  | 11c | Strategies to improve adherence to intervention protocols, and any procedures for monitoring adherence (eg, drug tablet return, laboratory tests) | 13, 20-21 |  |  |
|  | 11d | Relevant concomitant care and interventions that are permitted or prohibited during the trial | none |  |  |
| Outcomes | 12 | Primary, secondary, and other outcomes, including the specific measurement variable (eg, systolic blood pressure), analysis metric (eg, change from baseline, final value, time to event), method of aggregation (eg, median, proportion), and time point for each outcome. Explanation of the clinical relevance of chosen efficacy and harm outcomes is strongly recommended | 6-7 |  |  |
| Participant timeline | 13 | Time schedule of enrolment, interventions (including any run-ins and washouts), assessments, and visits for participants. A schematic diagram is highly recommended (see Figure) | 6, 14-15 |  |  |
| Sample size | 14 | Estimated number of participants needed to achieve study objectives and how it was determined, including clinical and statistical assumptions supporting any sample size calculations | 9 |  |  |
| Recruitment | 15 | Strategies for achieving adequate participant enrolment to reach target sample size | 8-9 |  |  |
| **Methods: Assignment of interventions (for controlled trials)** | | |  |  |  |
| Allocation: |  |  |  |  |  |
| Sequence generation | 16a | Method of generating the allocation sequence (eg, computer-generated random numbers), and list of any factors for stratification. To reduce predictability of a random sequence, details of any planned restriction (eg, blocking) should be provided in a separate document that is unavailable to those who enrol participants or assign interventions | 9-10 |  |  |
| Allocation concealment mechanism | 16b | Mechanism of implementing the allocation sequence (eg, central telephone; sequentially numbered, opaque, sealed envelopes), describing any steps to conceal the sequence until interventions are assigned | 9-10 |  |  |
| Implementation | 16c | Who will generate the allocation sequence, who will enrol participants, and who will assign participants to interventions | 9-10 |  |  |
| Blinding (masking) | 17a | Who will be blinded after assignment to interventions (eg, trial participants, care providers, outcome assessors, data analysts), and how | 10-11 |  |  |
|  | 17b | If blinded, circumstances under which unblinding is permissible, and procedure for revealing a participant’s allocated intervention during the trial | n |  |  |
| **Methods: Data collection, management, and analysis** | | |  |  |  |
| Data collection methods | 18a | Plans for assessment and collection of outcome, baseline, and other trial data, including any related processes to promote data quality (eg, duplicate measurements, training of assessors) and a description of study instruments (eg, questionnaires, laboratory tests) along with their reliability and validity, if known. Reference to where data collection forms can be found, if not in the protocol | 13-16 |  |  |
|  | 18b | Plans to promote participant retention and complete follow-up, including list of any outcome data to be collected for participants who discontinue or deviate from intervention protocols | 20-21 |  |  |
| Data management | 19 | Plans for data entry, coding, security, and storage, including any related processes to promote data quality (eg, double data entry; range checks for data values). Reference to where details of data management procedures can be found, if not in the protocol | 20 |  |  |
| Statistical methods | 20a | Statistical methods for analysing primary and secondary outcomes. Reference to where other details of the statistical analysis plan can be found, if not in the protocol | 19-20 |  |  |
|  | 20b | Methods for any additional analyses (eg, subgroup and adjusted analyses) | 19-20 |  |  |
|  | 20c | Definition of analysis population relating to protocol non-adherence (eg, as randomised analysis), and any statistical methods to handle missing data (eg, multiple imputation) | 19-20 |  |  |
| **Methods: Monitoring** | | |  |  |  |
| Data monitoring | 21a | Composition of data monitoring committee (DMC); summary of its role and reporting structure; statement of whether it is independent from the sponsor and competing interests; and reference to where further details about its charter can be found, if not in the protocol. Alternatively, an explanation of why a DMC is not needed | 1 |  |  |
|  | 21b | Description of any interim analyses and stopping guidelines, including who will have access to these interim results and make the final decision to terminate the trial | no |  |  |
| Harms | 22 | Plans for collecting, assessing, reporting, and managing solicited and spontaneously reported adverse events and other unintended effects of trial interventions or trial conduct | 15, 20-21 |  |  |
| Auditing | 23 | Frequency and procedures for auditing trial conduct, if any, and whether the process will be independent from investigators and the sponsor | 20-21 |  |  |
| Ethics and dissemination | | |  |  |  |
| Research ethics approval | 24 | Plans for seeking research ethics committee/institutional review board (REC/IRB) approval | 20-21 |  |  |
| Protocol amendments | 25 | Plans for communicating important protocol modifications (eg, changes to eligibility criteria, outcomes, analyses) to relevant parties (eg, investigators, REC/IRBs, trial participants, trial registries, journals, regulators) | 21 |  |  |
| Consent or assent | 26a | Who will obtain informed consent or assent from potential trial participants or authorised surrogates, and how (see Item 32) | 9, 20 |  |  |
|  | 26b | Additional consent provisions for collection and use of participant data and biological specimens in ancillary studies, if applicable |  |  |  |
| Confidentiality | 27 | How personal information about potential and enrolled participants will be collected, shared, and maintained in order to protect confidentiality before, during, and after the trial | 20-21 |  |  |
| Declaration of interests | 28 | Financial and other competing interests for principal investigators for the overall trial and each study site | 1 |  |  |
| Access to data | 29 | Statement of who will have access to the final trial dataset, and disclosure of contractual agreements that limit such access for investigators | 20 |  |  |
| Ancillary and post-trial care | 30 | Provisions, if any, for ancillary and post-trial care, and for compensation to those who suffer harm from trial participation | 20 |  |  |
| Dissemination policy | 31a | Plans for investigators and sponsor to communicate trial results to participants, healthcare professionals, the public, and other relevant groups (eg, via publication, reporting in results databases, or other data sharing arrangements), including any publication restrictions | 21 |  |  |
|  | 31b | Authorship eligibility guidelines and any intended use of professional writers | 1 |  |  |
|  | 31c | Plans, if any, for granting public access to the full protocol, participant-level dataset, and statistical code | 1 |  |  |
| Appendices |  |  |  |  |  |
| Informed consent materials | 32 | Model consent form and other related documentation given to participants and authorised surrogates | Y |  |  |
| Biological specimens | 33 | Plans for collection, laboratory evaluation, and storage of biological specimens for genetic or molecular analysis in the current trial and for future use in ancillary studies, if applicable | 16 |  |  |

*It is strongly recommended that this checklist be read in conjunction with the SPIRIT 2013 Explanation & Elaboration for important clarification on the items. Amendments to the protocol should be tracked and dated. The SPIRIT checklist is copyrighted by the SPIRIT Group under the Creative Commons “[Attribution-NonCommercial-NoDerivs 3.0 Unported](http://www.creativecommons.org/licenses/by-nc-nd/3.0/)” license.

**Supplementary file 2: Efficacy of Zinc Fortified and Fermented Wheat Flour (EZAFFAW): Randomized Controlled Trial Protocol**

**INTRODUCTION**

Zinc is integral to numerous essential metabolic pathways and usually manifests as a rather nonspecific deficiency, with varying severity and varies by age (1). In childhood, zinc deficiency appears as growth retardation and cognitive impairment (2, 3), recurrent infections including diarrhea (4), loss of hair, conjunctival and eyelid inflammation. In adolescents and adults, zinc deficiency can lead to fertility issues, reduced work capacity and metabolic disorders (5, 6) whereas for the elderly population, recurrent infections are a common manifestation (7).

Zinc supplementation, administered in pharmacological doses via pills or syrups, has demonstrated several positive effects in different age groups. In children, zinc supplementation suggests improvement in mean serum zinc concentration and a modest reduction in overall childhood mortality (8, 9). Zinc supplementation also reduces the incidence of diarrhea and its morbidity but has shown no effect on pneumonia and malaria morbidity (10-12) and evidence also suggests a small improvement in height with zinc supplementation in children (8, 9). There is no convincing evidence for the effectiveness of zinc supplementation on pregnant women except for a small effect on preterm births in low- and middle-income countries (13). In adults with insulin resistance, zinc supplementation does not appear to prevent the onset of type 2 diabetes (14), while zinc supplementation for individuals aged 55-87 has shown to reduce the incidence of infections and improve plasma zinc concentration (15). The potential impact of zinc on atherosclerotic disease remains inconclusive, despite its theoretical benefits in reducing plasma lipid peroxidation end products and endothelial cell adhesion molecules. (16). A recent meta-analysis suggests that low-dose, long-term zinc intake from supplements, and potentially biofortification, may offer benefits in terms of risk factors for type 2 diabetes (T2DM) and cardiovascular disease (CVD) (17).

Zinc bioavailability from a mixed or vegetarian diet based on refined cereal grains is estimated to be 26–34%, whereas 18–26% is absorbed from an unrefined cereal-based diet (18). The actual amount of absorbed zinc not only depends on the zinc content of the consumed diet but is highly affected by its intestinal zinc bio-accessibility and bio-availability (18). The inhibitory effect of phytate on zinc absorption is concentration-dependent and has shown to be more important than the phytate content of the product itself (19). Fermentation theoretically results in higher zinc bioavailability if a significant reduction of phytate is achieved.

Food fortification with zinc appears as an attractive public health strategy, with numerous programs initiated in developing countries as a cost-effective way to combat zinc deficiency. (1, 12). Systematic reviews evaluating zinc fortification indicate significant improvements in plasma zinc concentration, reduced prevalence of zinc deficiency, increased child weight, and enhanced short-term auditory memory. However, data for adolescents, pregnant women, and lactating women are limited (20). The Biofortified Zinc Flour to Eliminate Deficiency (BiZiFED) project aimed to investigate the impact of biofortification in alleviating zinc deficiency in Pakistan. The trial involved an average flour consumption of 224 g/day and provided an additional daily zinc intake of between 3.0 and 6.0 mg for white and whole grain flour, respectively. After 4 weeks, there was a notable increase in plasma zinc concentration, but no significant effect was observed after 8 weeks (21).

T2DM poses a major public health challenge, due to its increasing prevalence and complex complications. In populations with reduced growth and thin physiques, like in India, there is an elevated risk of truncal obesity and metabolic issues due to sedentary behavior and high-glycemic diets, exacerbated by low birth weight (22). Hyperglycemia is the primary driver of disease progression. Chronic glucose exposure affects erythrocyte membranes during their long lifespan, leading to structural and functional disruptions. Non-enzymatic protein glycation in erythrocyte membranes occurs in two phases: early glycation (Schiff bases and Amadouri compounds) and advanced glycation (advanced glycation end products or AGEs), which are associated with diabetic complications and can serve as diagnostic or progression markers. Lipid peroxidation and reduced enzyme pump activity also occur in erythrocyte membranes of diabetic patients. Exploring lipid rafts and erythrocyte membrane fatty acids can offer valuable tools for long-term metabolic monitoring and potentially reveal novel biomarkers for diabetes and its complications.

In Pakistan, common naan, a type of flatbread, is typically made from fermented maida (all-purpose low extraction) wheat flour, characterized by refined flour with minimal bran and germ, an extraction rate of less than 80%, low ash content (less than 0.7%), and approximately 1% fat. However, in southern Pakistan, households commonly use unfermented whole wheat "naan" and "roti" instead. These whole wheat flatbreads offer additional health benefits, making the findings applicable to advocacy and potential scale-up in this context.

Wheat samples from Pakistan were tested, with zinc levels ranging from 20-50mg/kg. The trial chose two samples: low zinc (20-25mg/kg) and high zinc content which is agronomically zinc biofortified (>35mg/kg) content, grown under collaborator supervision at the study site.

Whole wheat was selected as the preferred choice since it aligns with the community's customary practice of grinding their wheat or purchasing it from a local chakki. Therefore, the aim of this study is to assess the effect of fermented and unfermented wheat flour flatbread from agronomically zinc biofortified when compared to conventional low-zinc wheat flour flatbread and its impact on health including zinc status, anthropometric outcomes, risk of T2DM and morbidity on adolescent and adult females.

**MATERIALS AND METHODS**

**Objectives**

The objective of this trial is to assess the effect of whole wheat flatbread made from 'high zinc wheat' - agronomically biofortified (fermented and unfermented) or 'post-harvest zinc-fortified wheat flour' compared to 'low zinc' whole wheat flatbread on zinc status and metabolic health in adolescents (10-19 years) and adult women (20-40 years).

**Study Design**

This would be a four arm individually randomized, double-blind trial conducted in a rural district of Pakistan. The study will adhere to the guidelines outlined in the Consolidated Standards of Reporting Trials (CONSORT) for randomized controlled trials (23). The trial is registered at clinicaltrials.gov with number NCT06092515(24).

## Intervention

Participants will be randomly divided into four groups according to the four-arm design in a 1:1:1:1 ratio (See **Figure 1** for study groups).

- Group 1: will receive fermented high zinc - agronomically biofortified wheat (>35mg/kg) flour flatbread.
- Group 2: will receive unfermented high zinc agronomically biofortified wheat (>35mg/kg) flour flatbread.
- Group 3: will receive fortified (post-harvest) whole wheat (80mg/kg) flour flatbread.
- Group 4: will receive low zinc conventional whole (20-25 mg/kg) wheat flour flatbread.

**Figure 1:** Study Flow Diagram

The intervention duration will be six months, and participants will be provided with the respective ‘flatbread’ with a vegetable curry or pulse (*daal*) meal once a day for six days a week for six months This meal would be distributed at schools/colleges or community spaces during break or after the school is over at 130pm.

**Outcomes**

Primary:

- Serum zinc concentration
- Zinc deficiency
- HbA1C
- HOMA-IR (cut-off >2) (25)
- Lipid profile (total cholesterol, very low-density lipoprotein (VLDL), low-density lipoprotein (LDL), high-density lipoprotein (HDL); triglycerides (TGs))

Secondary:

- Body Mass Index (BMI) - Severe thinness, thinness, normal weight, overweight, obese
- Red blood cell membrane fatty acids concentrations
- Anemia
- Blood Pressure
- School attendance
- Morbidity – Diarrhea, Acute respiratory infection (ARI) etc.

Compliance:

- Number of days each participant had flatbread.
- Flatbread consumed (weight) each day.

**Eligibility Criteria**

The study will include adolescents (aged 10-19 both male and female) and adult women (aged 20-40). Participants would be eligible if they can consume wheat flour (no celiac disease), are not severely malnourished, pregnant, or lactating at enrollment, with no known chronic disease, and not enrolled in any other nutrition program or taking micronutrient supplements. Individuals planning to leave the study site during the study period will also not be eligible.

For the adult women, an additional eligibility criterion will be to assess their risk for T2DM. These risk factors were modified from an existing risk assessment scale, FINDRISC (Finnish Diabetes Risk Score) and Type 2 Diabetes Risk Test by American Diabetes Association (ADA) (26, 27). Adult women with any one of the following risk factors will be eligible to be enrolled in the study.

- BMI (>25kg/m^2^)
- Waist circumference (> 31 inches)
- Family history of diabetes
- HbA1c (>5.1%)
- FBG (>100mg/dl)
- Homeostatic Model Assessment of Insulin Resistance (HOMA-IR) (>2)

The HbA1c threshold for prediabetes are lower in Pakistan population compared with international guidelines, hence we have kept this as 5.1% (28).

**Study Site**

The study will be conducted in Mithi located in district Tharparkar in the province of Sindh, Pakistan. Tharparkar district is located about 320 kms east from the provincial capital Karachi (29), and has a total area of 19,637 km^2^ and a population of 1,647,036 (rural: 1,514,502; urban: 132,534) of which 53.4% are males and 46.4% are females (30). Tharparkar is an impoverished district of Sindh, characterized by a tropical desert climate. A staggering 87% of its population lives below the poverty line (31), due to limited economic opportunities and dependence on seasonal rains (32). Administratively, it is divided in seven Talukas and subdivided in 44 union councils (29). The district experiences erratic annual rainfall, sometimes as low as 100mm, leading to droughts (33). In Sindh about 58.8% of the adolescent girls are anemic of which 63.1% are from rural districts of Sindh, while 21.4% women aged 15-49 years old have zinc deficiency (1). Tharparkar has high food insecurity (1), with 60% of the children under five stunted, 33.3% wasted, 19.8% with both stunting and wasting and 40.4% of the non-pregnant women of reproductive age are underweight (1). The population of Mithi are predominantly vegetarians.

We have selected two high schools in Mithi where we would enroll adolescents and an elementary college, midwifery college and the neighboring community for adults 20-40 years of age. A list of current students from the respective school/colleges and individuals residing in the community (not attending school) will be collected through household line listing. All individuals on this list will undergo an eligibility assessment and will be individually randomized to the four groups.

We will obtain written informed consent from all eligible participants, and assent from participants under 18 years of age and consent from their caretakers. Upon receiving consent, the list of eligible adolescents and female adults will be randomly assigned to one of four groups, which would be blinded, and color coded as red, blue, green, and black.

**Sample size and Sampling strategy**

Sample Size Calculation

The study sample size was calculated based on an individually randomized four arm design on the primary outcomes of mean serum zinc levels and HbA1c. The mean HbA1c taken was 5.62% (SD 1.96) (34) and mean serum zinc was 79.5 µg/dL (SD 35.9) according to NNS-2018 (1). The sample size was determined with a power of 0.8 and alpha of 0.05 to detect a difference of at least 0.12 effect size in the mean serum zinc levels between groups and to detect a 0.2 effect size in the mean HbA1c levels with a dropout rate of 10%. The sample size calculated was 250 participants in each group, with 210 adolescents (aged 10-18 years) and 40 adult women (aged 20-40 years). Hence, a total of 1000 participants would be randomly allocated to each of the groups in a 1:1:1:1 ratio.

Randomization and allocation concealment

Individuals will be randomly assigned in the four groups (red, blue, green and black) through a computer-generated random number list. A statistician will assign the individuals into four groups by a computer-generated list and would then assign color codes (red, blue, green, black) without knowing which color belongs to which group. This randomization was stratified on gender and age (10-13, 14-19, 20-40) and adjusted for serum zinc, BMI, zinc deficiency and HbA1C.

An independent consultant from an international University (University of Sydney) would be responsible for assigning the intervention and control group to each color group and will not be involved in the trial, including recruitment, implementation or evaluation. The consultant would be responsible for generating and safeguarding the code and would be the only person communicating with the flour mill (Shoaib Corporation) and nutrition laboratory to ensure that the correct flour is being milled and packed in the right color bag for each batch.

This flour mill site (Saleh Pat) is located about 500km from the trial site and the flour would be transported fortnightly to the field site. Shoaib Corporation has set up a separate local mill (chakki) for the study and has the warehouse where the two different varieties of wheat are being stored. They would be responsible to mill, fortify (ZnO - 80mg/kg) and pack high zinc, fortified, and low zinc wheat/flour in 40kgs polypropylene sacks, corresponding to a specific color. All subsequent operations will be organized using these color codes, with the codes only being revealed at the conclusion of the trial. To ensure accuracy, fortnightly samples of color-coded products will be sent to the laboratory and the results will be shared with the consultant for confirmation.

Blinding

Participants, study personnel, and the outcome assessors will be kept unaware of the study groups. All aspects of the study, from randomization to implementation, data collection, and analysis, will be conducted using color codes assigned by an independent statistician. Color codes will be applied to wheat bags, ensuring packing, storage, and transportation in appropriately colored containers. Once participants are randomized, they will receive a laminated ID card with their photo, with the card's color indicating their assigned group to ensure that participants do not attempt to swap groups. A study kitchen is set up at the field site and has separate color-coded tandoors (ovens) for each group and would also have separate teams preparing these flatbreads who will wear color-coded aprons. Lunchboxes containing flatbread and curry will also be color-coded and packed at the study kitchen and transported to respective schools/colleges and designated community spaces.

**Wheat Cultivation and Product Development**

Wheat Cultivation

The cultivation of wheat to be used for this study was carried out at Saleh Pat by Shoaib Corporation and appropriate land was selected. Two cultivars, namely *Faisalabad-2008* and *TD-1* were selected by consultation with local experts. After harvesting, the wheat was homogenized for both the variants and stored in the warehouse under standard conditions. Agronomic biofortified was produced by zinc foliar application and was sprayed 2 times with ZnSO4⋅7H2O at 15 days interval at the heading stage to the *Faisalabad-2008*. The conventional low wheat variety was grown in the same conditions, but without spraying.

Product Development

The post-harvest fortified wheat flour was prepared by adding 80 ppm of zinc to 1kg of conventional low zinc variety. The fortification was performed in small batches every 15 days. Fortification was carried out in two steps, first by making a concentrated premix. Initially 3.2 g of Zinc oxide was mixed with 1kg of flour and subsequently added this premix to 19kg conventional wheat in a rotation mixer and mixed and then further 20kg of wheat flour was added to make 40kg bag.

Product development and sensory testing were conducted at the Bahauddin Zakariya University in Multan and the Mithi field site. Each product was developed at different extraction rates and variable fermentation times, considering local contextual factors. The whole wheat product of both varieties and two-hours fermented flatbread were deemed acceptable by the panelists during sensory testing and hence were chosen for the trial. The details would be published in a separate paper.

**INTERVENTION DELIVERY**

Flatbread preparation, and delivery

The flatbread and the vegetable/pulses curry will be prepared in a specific study kitchen established at the study site and professional catering staff will be hired to prepare food following standard guidelines. Four color-coded tandoor ovens will be set up, and the designated kitchen staff for each group will wear color-matched uniforms. The regular menu will feature flatbreads with a choice of vegetable curry or pulses, designed weekly and prepared in the study kitchen to ensure consistency.

High zinc fortified flour and low zinc wheat flour will be mixed separately with water, salt, oil in their respective color-coded utensils. The ingredients will be kneaded by hand to form soft dough. The dough will then be covered with a muslin cloth and the resting time of the dough will be kept identical for each of the produced flour portions (30 min) (Table 2.) For fermented flour, we will add 3.5g yeast /kg of flour and let the dough rest for 2hrs.

Table 2: Ingredients for flatbread

| Ingredients |  | Unit | Quantity | |
| --- | --- | --- | --- | --- |
|  |  |  | Fermented | Unfermented |
| Wheat flour |  | g | 1000 | 1000 |
| Active dry yeast |  | g | 3.50 | - |
| Iodized Salt |  | g | 4 | 4 |
| Ghee/cooking oil |  | g | 30 | 30 |
| Water for kneading the dough |  | ml | 300 | 300 |
| One ball of dough |  | g (±5g) | 125.00 | |
| Weight of one ready cooked flatbread |  | g (±5g) | 100.00 | |

Each ball will be weighed on a weighing machine to ensure that each ball has a weight of 120 grams. It will be transferred to a baking tray or round cushion lined and these will be used to place the flatbreads inside separate tandoors for each group. To allow for proper rising, flatbreads will be spaced adequately inside the tandoor.

Prepared flatbreads will be packed in color coded lunch boxes and each box will have two flatbreads (each flatbread divided in four equal parts) and curry under supervision. These boxes will then be transported to the targeted schools/colleges/community space and packing will ensure that the freshness of flatbread is maintained.

Procedure of serving flatbread/curry in schools

Field staff will deliver the respective lunchbox to the specific group in each school. The adolescents in different groups in a single school/college would be segregated and seated in separate marked rooms. The lunch boxes would be delivered after matching participants' specific IDs on the box with the student badges. The staff will also have extra flatbreads in color-coded bags and would give to individuals who would demand for more. The study team will record the amount of flatbread consumed by each participant for each day and data recorded electronically on an android app. Research staff and teachers will ensure each participant receives their designated lunch, record food consumption, and maintain order, ensuring that food is not being shared or taken home.

**DATA COLLECTION**

Baseline and endline measurements will be conducted at fixed time points, whereas for midpoint assessments, sampling scheme will be assessed by randomized, arm-based stratified selection so that at each of the three time-points, the three arms will be equally represented.

For all study visits, electronic app-based questionnaires will be used to guide personnel in conducting structured interviews with the study participants at baseline and endline. Data on 24-hr dietary recall will be collected in paper-based questionnaires. A summary of all monitoring and visit-specific forms will be used throughout the study.

**Hiring and Training**

All staff would be hired after a rigorous scrutiny process and a preference would be given to local people as they are most aware of the local customs and language. Upon recruiting, the research staff would undergo a comprehensive eight-day training program and a refresher training at midline and before endline assessments. The training will cover areas of line listing, mobile app data collection, anthropometric measurements, blood pressure measurements, blood sampling, food handling and ethical conduct and adherence to regulations. Kitchen staff will receive comprehensive training on handling wheat, flour, dough, flatbread preparation, weighing methods, hygiene, and food packing.

**Pilot testing**

Survey questionnaires will be pilot tested with a small group of participants (maximum 50) for response latency, question interpretation and appropriateness. Face validity and construct validity for all questionnaires will also be conducted with a small expert group with knowledge in public health and nutrition epidemiology.

**Baseline, Midline, and Endline Data Collection**

Participants will undergo assessments at baseline and endline, where we will gather data on socio-demographic factors, dietary intake via 24-hour dietary recall, anthropometric measurements (weight, height, MUAC, waist circumference), blood pressure measurements, and blood samples will be collected for biochemical analysis, including serum zinc levels, FBS, HbA1C, and insulin at both time points. Lipid profile and RBC membrane fatty acids will be assessed only at the endline. At midline, we will conduct 24-hour dietary recalls, measure serum zinc and HbA1C for a subset from each group. The data for morbidity (diarrhea and ARI) will be collected fortnightly for the entire duration of the intervention.

Demographic and socioeconomic indicators of households

Information on socio-economic status, gender, ethnicity, level of education, marital status, and occupation of the household head, water, sanitation, hygiene (WASH) and food insecurity will be collected. Household information will be captured from the head of the household or any knowledgeable member of the household (aged 18 years or more).

Dietary Intake

Dietary intake will be assessed using the USAID FANTA Household Dietary Diversity Scale (HDDS) and a 24-hour dietary recall questionnaire. The 24-hour recall will be conducted for the entire sample at baseline, midline, and endline, covering weekdays and weekends to capture dietary variability. Detailed food information will include description, additives, combinations, brand (if relevant), quantity, time, occasion, source, and consumption location (home or elsewhere). Interviewers and staff will be familiar with local foods, aided by guidance documents. Local utensils and portion-size photographs will assist in estimation.

Morbidity

Incidence and duration of diarrheal episodes and respiratory tract infections will be recorded every two weeks throughout the study.

Anthropometric measurements

Trained research staff will conduct anthropometric assessments at households and schools. Weight and height measurements will be recorded to the nearest 0.1 kilograms and centimeters, respectively. This will be done with participants in light clothing and without shoes, using a Seca digital floor scale (model 813) and Seca stadiometer (model 213). Mid-Upper Arm Circumference (MUAC) and waist circumference will be determined using standardized procedures and a MUAC measuring tape (Seca 201). All measurements will be taken in duplicate by two research staff members, and if discrepancies exceed 1 cm for height, 0.5 kg for weight, or 0.5 cm for MUAC/waist circumference, a third measure will be taken by the team leader and recorded using standardized procedures.

Biochemical sample collection

A certified phlebotomist will supervise blood collection. 23-mm gauge needles will be used for adolescents and women of reproductive age, with trace element-free tubes for blood collection. Used needles, sharps, and other consumables will be disposed-off safely. Strict personal hygiene will be maintained, including the use of disposable gloves and hand sanitizer. Skin cleaning and vein visibility will be ensured, and 5 ml of venous blood will be collected. Samples will be labelled with barcoded stickers. After labeling, the collection tubes will be left undisturbed for 30 minutes, and then centrifuged at 3000 rpm for 10 minutes. Serum (at least 1.0 ml) will be transferred to pre-labelled tubes and then placed in zip-lock bags. These serum samples will be stored in cool boxes at 2 to 8°C with the inclusion of ice packs. Used consumables will be safely disposed safelymulti speciality, .

**DATA ANALYSIS**

**Biochemical variables**

Analysis of samples will be conducted at Nutrition research lab, Aga Khan University, where external quality assurance (VITAL-EQA) is managed by Centers for Disease Control and Prevention (CDC). Individual biomarker concentrations will be used to determine deficiency using standard cut-offs for age and sex. CRP concentrations will be used to identify inflammation for all participants. We will check whether adjustment is necessary for serum zinc concentration and will be adjusted according to the BRINDA project guidelines to determine zinc deficiency (VAD) and if so present both adjusted and unadjusted values. Fasting blood glucose and blood Hb1C levels will be measured using latex agglutination inhibition and photometric assays, respectively on cobas c 311 biochemistry analyzer (Roche Diagnostics). Serum zinc levels will be measured using flame atomic absorption spectroscopy on Thermo Fisher iCE 3300 AAS Atomic Absorption Spectrometer.

**Anthropometric variables**

Anthropometric variables including the average height (m), weight (kg), waist circumference and MUAC would be collected in duplicate and the average (mean) of acceptable paired measures will be used in the analysis. Participant Body Mass Index (BMI) will be calculated and converted to BMI-for-age z-scores (BAZ) along with height-for-age z-scores (HAZ) according to chronological age using the WHO Growth Reference for Adolescents and WHO-package for R. Participants with a HAZ <-2 SD will be classified as stunted; severe thinness with a BAZ ≤ -3 SD; thinness with a BAZ > -3 SD to ≤-2 SD, normal weight with a BAZ >-2 SD to <+1SD, overweight with a BAZ >+1 SD to < +2 SD and, obese with a BAZ ≥+2 SD. The prevalence of anthropometric indicator categories will be reported as the proportion of participants who did not achieve the respective cut-off. MUAC will be assessed using cut-offs outlined in the Integrated Management of Adolescent and Adult Illness (WHO) as the proportion of participants with MUAC < 160 mm.

**Food Security and** **24-hour dietary recall**

Using the eight dichotomous questions (yes/no) within the Household FIES, households will be classified based on the total number of affirmative responses ranging from 0-8. As recommended by FAO, Rasch modelling techniques will be used (52).

The data collected from 24-hour recall will reveal the nutrient consumption patterns of each child beyond the study-provided food, aiding in assessing the actual intervention impact. Given the lack of a food composition database for Pakistan, we intend on using a database compiled by a recent study in Pakistan (MAL-ED) which compiled nutrient data from various sources. This comprehensive database includes foods from multiple references, including World Food Dietary Assessment System, USDA National Nutrient Database, NUTTAB online database, Composition of Foods Integrated Database, and the Food Composition Table for Bangladesh. If values could not be located through these sources, values were calculated using common recipes by either the PKN or INV sites of the MAL-ED study (35-45). The final contents of the MAL-ED Pakistan Food Composition Table consist of a combination of individual food items pulled from the sources referenced above, and nutrient values derived from calculating nutrients in the recipes collected as part of the study.

A trained nutritionist will assign food codes, and retention factors will be applied to account for nutrient changes due to different cooking methods. The mean total intake of each nutrient will be calculated using frequency weight and nutrient content for each food.

**Analysis**

This trial will adhere to CONSORT guidelines for randomized trials, with primary analysis on an intention-to-treat basis (23).

Both primary and secondary data will be analyzed following the same principle. Descriptive statistics will include mean and standard deviation for symmetrically distributed continuous variables, median and interquartile range for asymmetrically distributed ones, and frequency with percentages for categorical data. Associations between dependent and independent categorical variables will be accessed using chi-square test, while one-ANOVA will be used to assess difference in continuous outcomes by intervention groups. Significant association further assessed by post-hoc test adjusting for multiple comparisons. Multivariate analysis will be performed using linear or logistic regression to assess the impact of intervention adjusting for potential confounding factors including education of parents, household socioeconomic status, food insecurity and dietary intake.

**Data Management**

Data collection will be electronic, using Android OS devices with a customized Java application, incorporating data quality checks in real-time. Data will be transferred daily to the Aga Khan University (AKU) server via the internet or manually via USB in areas with no internet access. Password protection will restrict data access, with encryption and anonymization for confidentiality. An AKU repository will store data, accessible only by authorized personnel through AKU-LAN identification. A backup and fail-over server will ensure data security. Documentation including an installation guide, user manual, and database documentation will facilitate data transfer. Field supervisors will conduct spot checks, fortnightly refresher sessions, and monitor 10% of participants. Laboratory procedures will be quality-assessed through result rechecks and standardization.

**Ethical Considerations**

This study will secure ethical approval from both the Aga Khan University Ethical Review Committee (ERC) and the National Bioethics Committee (NBC), Pakistan. We will consistently uphold ethical principles, ensuring autonomy, anonymity, confidentiality, and equity throughout the study. Informed consent will be obtained from all eligible participants, with parental or caregiver consent for those under 18, alongside assent.

Collaborations with the Education and Health departments of the district will be closely maintained. Data collected will be anonymized and securely stored with robust data protection measures. Participants diagnosed with T2DM will be referred to appropriate health facilities. Participants will be free to withdraw from the trial at any point. We will conduct regular community engagement events and information sessions during the intervention to address concerns and encourage continued participation.

The safety of participants will be continuously monitored by study personnel, and in the event of any adverse incidents, affected individuals will be promptly referred to a healthcare facility for necessary treatment.

Ethical conduct with trial participants, especially children, will adhere to UNICEF's ethical guidelines (46). These principles emphasize the utmost respect for every child's dignity and rights, ensuring privacy, confidentiality, and participation in decisions that affect them.

**REFERENCES**

1. Shah D, Sachdev HS, Gera T, De-Regil LM, Pena-Rosas JP. Fortification of staple foods with zinc for improving zinc status and other health outcomes in the general population. Cochrane Database Syst Rev. 2016(6):Cd010697.

2. Gogia S, Sachdev HS. Zinc supplementation for mental and motor development in children. Cochrane Database Syst Rev. 2012;12:Cd007991.

3. Levenson CW, Morris D. Zinc and neurogenesis: making new neurons from development to adulthood. Adv Nutr. 2011;2(2):96-100.

4. Lazzerini M, Ronfani L. Oral zinc for treating diarrhoea in children. Cochrane Database Syst Rev. 2012(6):Cd005436.

5. Bernhardt ML, Kong BY, Kim AM, O'Halloran TV, Woodruff TK. A zinc-dependent mechanism regulates meiotic progression in mammalian oocytes. Biol Reprod. 2012;86(4):114.

6. Kawade R. Zinc status and its association with the health of adolescents: a review of studies in India. Glob Health Action. 2012;5:7353.

7. Pae M, Meydani SN, Wu D. The role of nutrition in enhancing immunity in aging. Aging Dis. 2012;3(1):91-129.

8. Mayo-Wilson E, Junior JA, Imdad A, Dean S, Chan XH, Chan ES, et al. Zinc supplementation for preventing mortality, morbidity, and growth failure in children aged 6 months to 12 years of age. Cochrane Database Syst Rev. 2014(5):Cd009384.

9. Brown KH, Peerson JM, Baker SK, Hess SY. Preventive zinc supplementation among infants, preschoolers, and older prepubertal children. Food Nutr Bull. 2009;30(1 Suppl):S12-40.

10. Yakoob MY, Theodoratou E, Jabeen A, Imdad A, Eisele TP, Ferguson J, et al. Preventive zinc supplementation in developing countries: impact on mortality and morbidity due to diarrhea, pneumonia and malaria. BMC Public health. 2011;11(3):1-10.

11. Penny ME. Zinc supplementation in public health. Annals of Nutrition and Metabolism. 2013;62(Suppl. 1):31-42.

12. Das JK, Kumar R, Salam RA, Bhutta ZA. Systematic review of zinc fortification trials. Annals of Nutrition and Metabolism. 2013;62(Suppl. 1):44-56.

13. Ota E, Mori R, Middleton P, Tobe-Gai R, Mahomed K, Miyazaki C, et al. Zinc supplementation for improving pregnancy and infant outcome. Cochrane Database Syst Rev. 2015(2):CD000230.

14. El Dib R, Gameiro OL, Ogata MS, Modolo NS, Braz LG, Jorge EC, et al. Zinc supplementation for the prevention of type 2 diabetes mellitus in adults with insulin resistance. Cochrane Database Syst Rev. 2015(5):CD005525.

15. Prasad AS, Beck FW, Bao B, Fitzgerald JT, Snell DC, Steinberg JD, et al. Zinc supplementation decreases incidence of infections in the elderly: effect of zinc on generation of cytokines and oxidative stress. Am J Clin Nutr. 2007;85(3):837-44.

16. Prasad AS. Discovery of human zinc deficiency: its impact on human health and disease. Adv Nutr. 2013;4(2):176-90.

17. Pompano LM, Boy E. Effects of dose and duration of zinc interventions on risk factors for type 2 diabetes and cardiovascular disease: a systematic review and meta-analysis. Advances in Nutrition. 2021;12(1):141-60.

18. International Zinc Nutrition Consultative Group (IZiNCG); Brown KH, Rivera JA, Bhutta Z, Gibson RS, King JC, Lönnerdal B, Ruel MT, Sandtröm B, Wasantwisut E, Hotz C. International Zinc Nutrition Consultative Group (IZiNCG) technical document #1. Assessment of the risk of zinc deficiency in populations and options for its control. Food Nutr Bull. 2004 Mar;25(1 Suppl 2):S99-203. PMID: 18046856.

19. Oberleas D, Harland BF. Phytate content of foods: effect on dietary zinc bioavailability. J Am Diet Assoc. 1981 Oct;79(4):433-6. PMID: 7288050.

20. Tsang BL, Holsted E, McDonald CM, Brown KH, Black R, Mbuya MN, et al. Effects of foods fortified with zinc, alone or cofortified with multiple micronutrients, on health and functional outcomes: a systematic review and meta-analysis. Advances in Nutrition. 2021;12(5):1821-37.

21. Lowe NM, Zaman M, Khan MJ, Brazier AK, Shahzad B, Ullah U, et al. Biofortified Wheat Increases Dietary Zinc Intake: A Randomised Controlled Efficacy Study of Zincol-2016 in Rural Pakistan. Frontiers in nutrition. 2021;8.

22. Wells JC, Pomeroy E, Walimbe SR, Popkin BM, Yajnik CS. The elevated susceptibility to diabetes in India: an evolutionary perspective. Frontiers in public health. 2016 Jul 7;4:145.

23. Moher D, Hopewell S, Schulz KF, Montori V, Gøtzsche PC, Devereaux PJ, et al. CONSORT 2010 explanation and elaboration: updated guidelines for reporting parallel group randomised trials. International journal of surgery. 2012;10(1):28-55.

24. ClinicalTrials.gov 2023 [Available from: <https://register.clinicaltrials.gov/prs/app/action/SelectProtocol?sid=S000DNYW&selectaction=Edit&uid=U00043LM&ts=2&cx=clyujg>.

25. Majid H, Masood Q, Khan AH. Homeostatic model assessment for insulin resistance (HOMA-IR): a better marker for evaluating insulin resistance than fasting insulin in women with polycystic ovarian syndrome. J Coll Physicians Surg Pak. 2017;27(3):123-6.

26. Janghorbani M, Adineh H, Amini M. Evaluation of the Finnish Diabetes Risk Score (FINDRISC) as a screening tool for the metabolic syndrome. The review of diabetic studies: RDS. 2013;10(4):283.

27. American Diabetes Association. 60-Second Type 2 Diabetes Risk Test 2022 [Available from: <https://www.diabetes.org/risk-test>.

28. Basit A, Fawwad A, Abdul Basit K, et al. Glycated hemoglobin (HbA1c) as diagnostic criteria for diabetes: the optimal cutoff points values for the Pakistani population; a study from second National Diabetes Survey of Pakistan (NDSP) 2016–2017. BMJ Open Diab Res Care 2020;8:e001058. doi:10.1136/bmjdrc-2019-001058.

29. United Nations Children's Fund, Government of Pakistan. Nutrition And Mortality Survey.; 2014.

30. Pakistan Bureau of Statistics (PBS). 6th Population & Housing Census 2017. 2017 [Available from: <https://www.pbs.gov.pk/content/final-results-census-2017>.

31. Planning Commission of Pakistan, UNDP, Oxford Poverty and Human Development Initiative. Multidimensional Poverty in Pakistan. 2014.

32. Talpur MA, Mari SA. Seasonal Migration in Tharparkar District of Sindh Province, Pakistan: An In-depth Empirical Analysis. Pak J App Eco. 2020.

33. Kunbher AD, Ullah S, Alam M. Multi-sector, nutrition-sensitive response to drought emergency in Pakistan. Field Exchange 55. 2017:98.

34. Aamir AH, Ul-Haq Z, Mahar SA, Qureshi FM, Ahmad I, Jawa A, et al. Diabetes Prevalence Survey of Pakistan (DPS-PAK): prevalence of type 2 diabetes mellitus and prediabetes using HbA1c: a population-based survey from Pakistan. BMJ open. 2019;9(2):e025300.

35. U.S. Department of Agriculture, Agricultural Research Service. 2007. USDA Table of Nutrient Retention Factors, Release 6.

36. U.S. Department of Agriculture, Agricultural Research Service. 2014. USDA National Nutrient Database for Standard Reference, Release 27. Nutrient Data Laboratory Home Page, <http://www.ars.usda.gov/ba/bhnrc/ndl>.

37. U.S. Department of Agriculture, Agricultural Research Service. 1975. Agricultural Handbook no. 102; Food yields summarized by different stages of preparation. Washington, D.C.

38. FAO. Food composition: Overview of the WorldFood Dietary Assessment System. Available at: <http://www.fao.org/infoods/software_overview_en.stm>.

39. Bognar A. 2002. Tables on weight yield of food and retention factors of food constituents for the calculation of nutrient composition of cooked foods (dishes). .

40. Bognar A. 1998. Comparative study of frying to other cooking techniques influence on the the nutritive value. Grasas y Aceites 49;3-4. .

41. Food Composition Table for Bangladesh - Nazma Shaheen, Abu Torab MA Rahim, Md. Mohiduzzaman, Cadi Parvin Banu, Md. Latiful Bari, Avonti Basak Tukun, MA Mannan, Lalita Bhattacharjee, Barbara Stadlmayr. Institute of Nutrition and Food Science; Centre for Advanced Research in Sciences; University of Dhaka, Dhaka-1000, Bangladesh. 2013 (English).

42. NUTTAB 2010 online searchable database ([www.foodstandards.gov.au](file:///C:\Users\jai.das\Downloads\www.foodstandards.gov.au)).

43. McCance and Widdowson's The Composition of Foods Integrated Dataset (CoF IDS), 2002.

44. Goplan, C., Sastri, B.V. and Balasubramnian, S.C. (1981), Nutritive Values of Indian Foods, National Institute of Nutrition, Indian Council of Medical Research, Hyderabad, New Delhi.

45. I.J. Alinnor and C.O. Akalezi, 2010. Proximate and Mineral Compositions of Dioscorea rotundata (White Yam) and Colocasia esculenta (White Cocoyam). Pakistan Journal of Nutrition, 9: 998-1001.

46. United Nations Children's Fund. Ethical guidelines for reporting on children: UNICEF; 2022 [Available from: <https://www.unicef.org/montenegro/en/ethical-guidelines-reporting-children>.
