# Supplementary material for: Efficacy of Zinc Fortified and Fermented Wheat Flour (EZAFFAW): A randomized controlled trial protocol
Source: PLoS One. 2024 Jun 20;19(6):e0304462. doi: 10.1371/journal.pone.0304462 (PMC11189184; doi:10.1371/journal.pone.0304462)
Supplement: Attachment — Submitted filename: responses_comments.docx [file pone.0304462.s002.docx]

Reviewer #1: Thank you for the opportunity to review this paper. This is an interesting manuscript presenting a protocol of a randomized controlled trial on efficacy of Zinc Fortified and fermented wheat flour. My review mainly concerns only the statistical aspects of the study. Some questions reported below were raised and in my view, it is not acceptable in this form for the publication in this journal. The "trial analysis" paragraph should be more detailed. Particularly in relation to multivariate models. Authors should anticipate Intention-to-treat analysis of the missing data and possible sensitivity analyses.

**Response:** we would like to thank the reviewers for their review and encouragement and as suggested, we have added the required details in the data analysis section.

Reviewer #2: Upon reviewing the manuscript titled "Efficacy of Zinc Fortified and Fermented Wheat Flour (EZAFFAW): A Randomized Controlled Trial Protocol" (Manuscript Number: PONE-D-23-42882), it became evident that the authors have outlined a comprehensive and timely protocol that addresses the urgent need of developing strategies to mitigate zinc deficiency among the population, particularly adolescent and young women living in economically disadvantages areas. The proposed protocol includes 1000 participants, allocated by stratified randomization to four groups. Zn supplementation of the daily diet via its incorporation into bread, a common food for the population appears to be a promising option.

Monitoring the impacts on morbidity, anthropometric and biochemical indicators, and other key metrics is ensured through the evaluation of multiple indicators.

The processes for obtaining flour that has been fortified with ZnO or biofortified via foliar spraying with ZnSO4.7H2O is comprehensively elucidated, along with the process of bread preparation. Nonetheless, I would like to pose a few questions:

1. Why was not the same variety of wheat used for the production of both agronomically biofortified and biofortified flour?

**Response:** we would like to thank the reviewers for the thorough review and comments. We liaised with various experts and as Akber-19 has been introduced as a zinc enriched variety and locally developed, so we wanted to compare this against a conventional variety and hence chose to use two different varieties.

2. During the preparation of the bread, the dough was left to rest for 30 minutes or more (depending on whether it was fermented or not) at what temperature?

**Response:** thanks, and we have now added this.

3. In the section "Data analysis - Food testing" the quantification of phytate includes a stirring period of 3 hours. Regarding the technique and temperature involved, I propose that it be clarified more deeply.

**Response:** we have now added the details here.
